# Colloid osmotic pressure of contemporary and novel transfusion products

**DOI:** 10.1111/vox.12932

**Published:** 2020-05-06

**Authors:** Robert B. Klanderman, Joachim J. Bosboom, Herbert Korsten, Thomas Zeiler, Ruben E. A. Musson, Denise P. Veelo, Bart. F. Geerts, Robin van Bruggen, Dirk de Korte, Alexander P. J. Vlaar

**Affiliations:** ^1^ Department of Intensive Care Amsterdam UMC University of Amsterdam Amsterdam The Netherlands; ^2^ Laboratory of Experimental Intensive Care and Anesthesiology Amsterdam UMC University of Amsterdam Amsterdam The Netherlands; ^3^ Department of Anesthesiology Amsterdam UMC University of Amsterdam Amsterdam The Netherlands; ^4^ Department of Product and Process Development Sanquin Blood Bank Amsterdam The Netherlands; ^5^ German Red Cross Blood Service West Hagen Germany; ^6^ Laboratory for Clinical Chemistry and Haematology University Medical Centre Utrecht Utrecht The Netherlands; ^7^ Department of Blood Cell Research Sanquin Research and Landsteiner Laboratory Amsterdam UMC University of Amsterdam Amsterdam The Netherlands

**Keywords:** blood components, blood safety, plasma, platelet transfusion, red cell components, transfusion medicine (in general)

## Abstract

**Background and Objectives:**

Colloid osmotic pressure (COP) is a principal determinant of intravascular fluid homeostasis and a pillar of fluid therapy and transfusion. Transfusion‐associated circulatory overload (TACO) is a leading complication of transfusion, and COP could be responsible for recruiting additional fluid. Study objective was to measure COP of blood products as well as investigate the effects of product concentration and storage lesion on COP.

**Materials and Methods:**

Three units of each product were sampled longitudinally. COP was measured directly as well as the determinants thereof albumin and total protein. Conventional blood products, that is red blood cell (RBC), fresh‐frozen plasma (FFP) and platelet concentrates (PLTs), were compared with their concentrated counterparts: volume‐reduced RBCs, hyperconcentrated PLTs, and fully and partially reconstituted lyophilized plasma (prLP). Fresh and maximally stored products were measured to determine changes in protein and COP. We calculated potential volume load (PVL) to estimate volume recruited using albumin's water binding per product.

**Results:**

Colloid osmotic pressure varies widely between conventional products (RBCs, 1·9; PLTs, 7·5; and FFP, 20·1 mmHg); however, all are hypooncotic compared with human plasma COP (25·4 mmHg). Storage lesion did not increase COP. Concentrating RBCs and PLTs did not increase COP; only prLP showed a supraphysiological COP of 47·3 mm Hg. The PVL of concentrated products was lower than conventional products.

**Conclusion:**

Colloid osmotic pressure of conventional products was low. Therefore, third‐space fluid recruitment is an unlikely mechanism in TACO. Concentrated products had a lower calculated fluid load and may prevent TACO. Finally, storage did not significantly increase oncotic pressure of blood products.

## Introduction

Colloid osmotic pressure (COP) and its use in patient care is a topic that has been extensively investigated in previous years [1]. Measurement of COP in clinical practice has been abandoned as correction of low oncotic pressure did not lead to better patient outcomes [2, 3, 4, 5]. Nevertheless, plasma COP plays an important role in the clinicians' choice of fluid resuscitation and/or transfusion strategy, [6] and it might play a central role in transfusion‐associated circulatory overload (TACO) – the leading cause of transfusion‐related mortality [7, 8, 9].

Current understanding of TACO is that the volume of blood products infused overloads the heart and circulation [10, 11]. It is currently unclear how the pathogenesis of TACO is different to conventional fluid (e.g. crystalloid) overload as the incidence of TACO differs depending on the type of blood product transfused. Plasma appears most likely to induce TACO followed by red blood cell (RBC) concentrate products and platelet concentrates (PLTs) [12, 13, 14]. Moreover, observational data suggest larger volumes of crystalloid are required to induce circulatory overload compared with transfusion in addition to fluids [15].

Colloid osmotic pressure is likely to contribute to the development of circulatory overload. According to Starling's principles, colloidal or oncotic pressure is the net force, exerted by plasma bound proteins, attracting water from the interstitium into the capillaries [16]. This force directly counterbalances capillary hydrostatic pressure which drives water across the membrane of the vessels. While the revised Starling principles ascribe more importance to the endothelial barrier and glycocalyx, the fundamental driving pressure of COP and hydrostatic pressure still stand [16, 17]. As opposed to crystalloids, transfusion of blood products which contain protein along with osmotically active preservatives (e.g. mannitol) will raise COP, potentiating fluid recruitment from peripheral tissues and thereby increasing intravascular volume by more than the volume of product infused.

Advancements in blood banking practice have led to dramatic changes in transfusion product composition over the last decades, including universal leucoreduction and use of new storage solutions [18, 19]. The last study investigating COP in blood products dates back to 1993 [20], and reanalysis of contemporary products is warranted. Moreover, volume‐reduced blood products have recently become available and have been suggested for use in patients at risk of TACO [10, 21]. Volume‐reduced red blood cells (VR‐RBCs) have been used for years in neonatal and paediatric patients to limit volume load [22]. Volume‐reduced platelets (VR‐PLTs) are now available limiting the volume of one unit of platelets to 20 ml [23]. Finally, lyophilized plasma (LP) can be partially reconstituted in half the original volume (though this is off‐label use) to limit the volume of a plasma unit [24, 25]. However, if COP is a primary determinant, then reducing product volume while protein load is equal or increases will not prevent TACO.

Whether storage of blood products results in worse patient outcomes has been investigated in numerous studies [26]. Storage lesion is the time‐dependent degradation of blood products during preservation. Transfusion products containing cellular components, that is red blood cell and platelets, have an active metabolism, utilizing nutrients, producing lactate and a fraction lyses over time influencing cell‐free protein levels [27, 28]. While cells are not osmotically active, release of cell‐bound proteins can potentially increase COP, and this effect has to our knowledge never been investigated.

We hypothesized that the COP of transfusion products results in fluid recruitment from peripheral tissues leading to circulatory overload; the first step is to investigate these characteristics in vitro. We aimed to measure the COP of contemporary as well as novel blood products through direct and indirect measurements and calculate the blood volume changes related to the COP of transfusion products. Finally, we determined the effect of storage time on the COP in cellular blood products to investigate whether storage lesion contributes to TACO.

## Materials and methods

All blood products were supplied by Sanquin, the national Dutch blood bank, with the exception of LyoPlas, which was supplied by the German Red Cross Service West, Hagen, Germany. Volunteers provide informed consent prior to each donation, allowing for anonymous use of blood products for scientific purposes. Three individual units were investigated of each product listed in Supplementary Table S1.

### Red blood cell products

Products were randomly picked from donations processed at the blood bank on the day of testing. Briefly, donors and blood products were screened according to national guidelines. Red blood cells (RBCs) were produced from whole blood donations in a solution of citrate, phosphate and dextrose. After overnight storage, whole blood units were centrifuged and separated using the Compomat G5 (Fresenius Kabi – Bad Homburg, Germany) into plasma, buffy coat (to be used for platelet production) and erythrocytes. After addition of a storage solution of saline, adenine, glucose and mannitol, the RBC component was passed through a leucoreduction filter. Volume‐reduced red blood cells (VR‐RBCs) were made from freshly prepared RBC units, recentrifuged to remove most of the storage solution and plasma component after which 0·9% sodium chloride was added until a haematocrit of 75‐80% was reached. The maximum storage duration at 2–6°C of RBCs is 35 days. VR‐RBCs have limited nutrients and buffer to prevent degradation and Dutch national guidelines dictate a storage duration to a maximum of 6 h at 2–6°C.

### Platelet products

A single platelet concentrate unit (PLT) is produced from pooling the buffy coats of five donations, to which is added platelet additive solution C [PAS‐C, containing citrate, acetate and phosphate in sodium chloride (Fresenius Kabi)]. This resulted in a final platelet resuspension medium which still contains a residual volume of plasma (roughly 35% plasma and 65% PAS‐C). Buffy coat pools were centrifuged and separated into a waste product and a platelet concentrate (PLT) which was leucoreduced. Volume‐reduced or hyperconcentrated platelets (VR‐PLTs) were prepared from fresh PLT units. Platelet activation was inhibited by addition of 10% (v/v) anticoagulant‐citrate‐dextrose solution, solution A (ACD‐A), and centrifuged. The platelet‐poor plasma was removed after centrifugation, and the platelet component was resuspended in a volume of about 20 ml using a pre‐ACD‐A‐treated sample of the same product reserved for this purpose. PLT units can be stored at 20–24°C for 7 days maximum and VR‐PLTs can be stored for to a maximum of 6 h, as the high platelet concentration results in rapid lactate accumulation and acidification [23].

### Plasma products

Fresh‐frozen plasma (FFP) was acquired from donated whole blood. After leucoreduction, whole blood was centrifuged and automatically separated using the Compomat G5 (Fresenius Kabi) to obtain leucocyte‐depleted plasma. Plasma units were randomly chosen from the Dutch blood bank storage and underwent one freeze–thaw cycle for sampling purposes. Lyophilized plasma (LP) was used from the commercially available stock (LyoPlas N‐W®Germany Red Cross Blood Service West – Hagen, Germany). Partially reconstituted LP and fully reconstituted LP were sampled from the same product, to prevent inter‐unit variation as a confounder. Initially, a partially reconstituted solution in 50% of original volume (2x concentrated) was prepared (LP50%). Aside from the partial reconstitution, manufacturers' guidelines were followed. Under gentle agitation, achieved through a tube rotator, 100 ml out of the normally 200 ml of water for injection was added to resuspend the freeze‐dried product. A sample of 20·0 ml was taken, and the residual 80·0 ml of LP50% was fully reconstituted (LP100%) with an additional 80·0 ml of water for injection. As plasma products lack a cellular component with ongoing metabolic activity, storage lesion was not evaluated.

### Colloid products

Human albumin 40 and 200 g/l were sampled from three different batches (Albuman®, Sanquin, Amsterdam, the Netherlands). Of hydroxyethyl starch 130/0·4, three samples were measured for reference (Tetraspan 6%, *B.*Braun, Melsungen, Germany).

### Sample collection and laboratory analysis

Samples were collected from each individual product unit after preparation at baseline (fresh) and after their respective maximum storage duration. Immediately after sampling, blood gas analysis and cell counting were performed using a RAPIDLab 1265 blood gas analyzer and an ADVIA 2120i (Siemens, Munich, Germany). Red blood cell and platelet products were centrifuged (2000 ***g*** for 10 min at 4°C), and supernatant from these products, as well as samples from non‐cellular products, was aliquoted and stored at −80·0°C to be analysed at a later time.

Direct COP was measured using an Osmometer 050 (Gonotec, Berlin, Germany). Measurements were performed at room temperature using a membrane colloid osmometer, with a membrane cut‐off permeability to molecules of 20 kDa or approximately 2·0 nm. The reference for endothelium permeability being approximately 3 nm in size [29]. Size of proteins of interest are all larger than the filter size and include albumin (7·5 nm), [30] RBC microparticles (100–1000 nm) [31] and free haemoglobin (5·0 nm) [32]. Indirect determinants of COP were also measured. Total protein and albumin were measured on an AU5811 clinical chemistry analyzer (Beckman Coulter, Brea, CA, USA). Total protein in samples with concentrations below the detection limit of clinical chemistry analysers was measured through a modified Lowry method. Albumin was determined using the bromocresol‐green method. Plasma osmolality was determined using an OSMO Station OM‐6050 (Arkray, Kyoto, Japan) using a freezing point method.

### Calculation of estimated colloid osmotic pressure

**Fig. 1:**
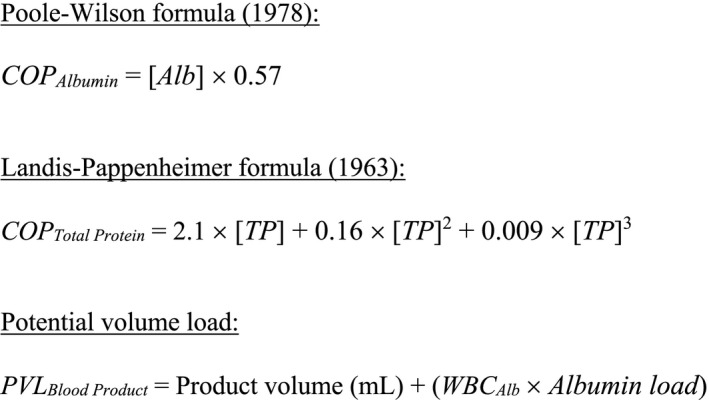
Calculating indirect colloid osmotic pressure and potential volume load. Abbreviations: Alb, albumin (g/l); TP, total protein (g/l); WBC_Alb_, water‐binding capacity of albumin (ml/g) is 18 ml/g; Albumin load = total amount of albumin in a single blood product (g/unit).

Different formulae have previously been used to calculate COP, and we applied the two most commonly used: (1) the Poole–Wilson formula using the plasma albumin concentration; and (2) the Landis–Pappenheimer formula using the plasma total protein concentration [33] Fig. 1.

### Study outcomes

Primary outcome is a descriptive analysis of COP of conventional and volume‐reduced blood products. Secondary outcomes include the calculated potential volume load, the correlation between total protein, albumin and osmolality as indirect measures of oncotic pressure compared with directly measured COP, and finally difference in COP as a result of storage.

### Statistical analysis

Descriptive characteristics are shown as mean and range. Changes in COP, and other biochemical determinants, due to storage were calculated using a paired *t*‐test, and correlations between indirect and direct measures of COP were assessed using Spearman's rank correlation test. Bland–Altman plots were generated for indirect measuring of COP compared with direct measurement. Potential volume load (PVL) was calculated per product using the water binding capacity for albumin (WBC_Alb_) of approximately 18 ml/g (Fig. 1.) [34].

## Results

**Fig. 2:**
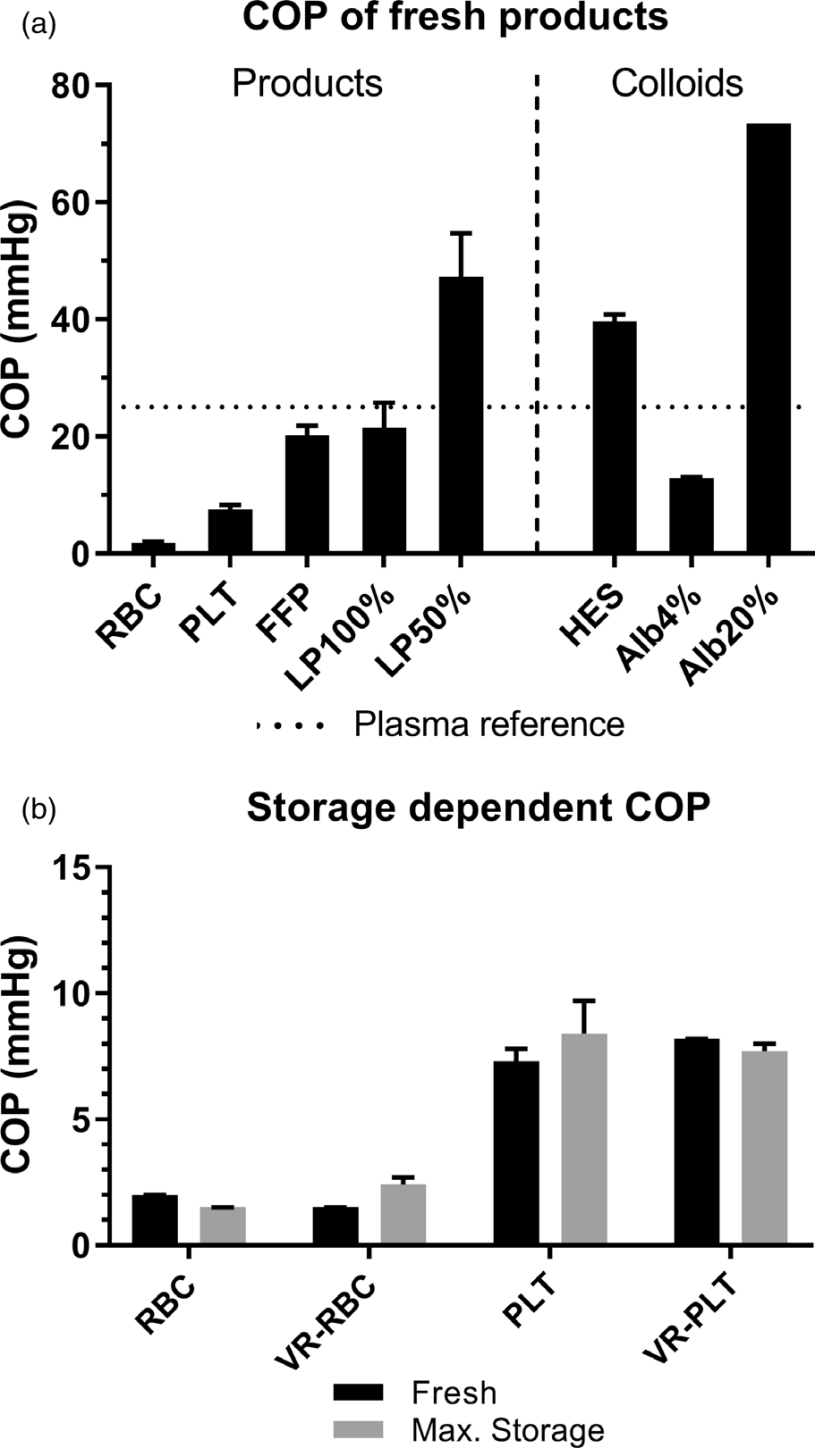
Colloid osmotic pressure of blood products. Panel (a): Colloid osmotic pressure of fresh products displayed as mean and range. Panel (b): Change in colloid osmotic pressure due to storage. Alb4%, albumin solution 4%; Alb20%, albumin 20%; COP, colloid osmotic pressure; FFP, fresh‐frozen plasma; HES, hydroxyethyl starch solution; LP50%, partially reconstituted lyophilized plasma; LP100%, fully reconstituted lyophilized plasma; PLT, platelet concentrate; RBC, red blood cell unit; VR‐PLT, volume‐reduced PLT; VR‐RBC, volume‐reduced RBC.

Colloid osmotic pressure of blood products has a wide variation with most notably a low oncotic pressure of RBC and PLTs units. Most standard plasma and plasma replacement products are hypooncotic, that is Alb4%, FFP and LP100, compared with human plasma COP (25·4 mm Hg–measured in supernatant of heparinized whole blood), with HES having a supraphysiologic COP of 39·6 ± 1·2 mm Hg (Fig. 2).

### Novel concentrated products vs. conventional products

There was minimal difference in COP between conventional RBCs vs. VR‐RBCs and PLTs vs. VR‐PLTs, respectively, −0·42 ± −0·04 mm Hg and 0·28 ± 0·03 mm Hg. Of concentrated blood products, partially reconstituted LP50% showed the greatest increase in COP. When comparing both Alb4% to Alb20%, and LP100% to LP50%, there is a non‐linear increase in COP (Table 1 *)*.

**Table 1:** Fresh blood product characteristics

Fresh blood product characteristics	Red blood cell products		Platelet products		Plasma products			Colloid products		
	RBC (*n* = 3)	VR‐RBC (*n* = 3)	PLT (*n* = 3)	VR‐PLT (*n* = 3)	FFP (*n* = 3)	LP100% (*n n* = 3)	LP50% (*n* = 3)	Alb4% (*n* = 3)	Alb20% (*n* = 3)	HES (*n* = 3)
COP (mm Hg)	1·9 (1·7–2·0)	1·5* (1·3–1·6)	7·5 (7·0–8·3)	7·8 (7·0–8·3)	20·1 (17·2–21·8)	21·5 (18·9–25·8)	47·3* (38·2–54·7)	12·8 (12·7–13·1)	73·5 (73·5–73·5)	39·6 (38·3–40·7)
Albumin (g/l)	<5·0	<5·0	13·1 (12·8–13·4)	12·9 (12·8–13·0)	34·8 (32·5–37·4)	36·1 (35·1–37·2)	58·8***, b (58·4–59·0)	41·2 (41·0–41·3)	203 (202–204)	‐
Total protein (g/l)	3·2 (2·6–4·3)	2·8 (2·4–3·3)	23·3 (23·0–24·0)	23·3 (22·0–23·0)	56·7 (52·0–60·0)	56·0 (55·0–58·0)	97·0***, b (96·0–99·0)	41·0 (41·0–41·0)	207·6 (207–208)	‐
Osmolality (mOsmol/l)	332 (332–334)	327**, a (327–329)	298 (275–299)	304 (304–305)	309 (307–310)	292 (290–294)	520 (520–523)	262 (262–263)	171 (171–171)	297 (296–297)
pH:	6·8 (6·8–6·9)	6·8 (6·8–6·9)	7·0 (7·0–7·0)	6·7 (6·7–6·7)	7·1 (7·1–7·1)	7·0 (7·0–7·0)	7·0 (7·0–7·0)	6·7 (6·7–6·7)	6·8 (6·7–6·8)	7·4 (7·4–7·4)
Glucose (mmol/l):	32·5 (32·5–33·8)	19·8 (19·7–20·2)	8·5 (8·5–8·8)	10·3 (10·2–10·6)	19·8 (19·5–20·8)	17·4 (16·9–17·5)	27·2 (26·5–27·6)	‐	‐	‐
Lactate (mmol/l):	4·33 (4·2–5·4)	5·0 (4·5–5·1)	3·4 (3·4–3·4)	5·0 (4·5–5·1)	<0·01	4·4 (4·3–4·7)	7·6 (7·2–7·9)	0·1 (0·1–0·2)	0·1 (0·01–0·1)	<0·01

Data are displayed as mean (range).

Difference in biochemical properties at maximum storage duration vs. fresh product tested using paired‐sample Student *t*‐test. Colloid products not tested.

* *P *< 0·05.

** *P *< 0·01.

*** *P *< 0·001.

### Potential for blood products to recruit fluid to the intravascular space

To determine whether products with a high COP are more prone to induce circulatory overload, a worst‐case estimated volume load per product was calculated. The absolute volume per product plus the volume of water potentially recruited by albumin was calculated as the potential volume load (PVL) – shown in Table 2. VR‐RBCs are least likely to result in absolute volume reduction of all novel volume‐reduced blood products, with VR‐PLTs estimated to have the largest volume reduction instead of conventional platelets resulting in a volume load of ±25 ml vs. 300 ml. The use of LP50% reduces the potential volume compared with FFP by approximately half.

**Table 2:** Absolute and recruited volume load reduction

Infusion product	Product volume	∆ Infused volume (ml, %)	Albumin load (g)	Potential volume load (ml, ∆%)^**^, ^a^	∆ Potential volume load (ml, %)^***^, ^b^
RBC:	±300 ml	−50·0 (−16·7%)	1·50^d^	327·0 (+9·0%)	−54·5 (−16·7%)
VR‐RBC:	±250 ml		1·25^d^	272·5 (+9·0%)	
PLT:	±300 ml	−280·0 (−93·3%)	3·93	370·7 (+23·6%)	−346·1 (−93·4%)
VR‐PLT:	20 ml		0·26	24·6 (+23·2%)	
FFP:	200 ml^c^		6·96	325·3 (+62·6%)	
LP100%:	200 ml	+0·0 (+0·0%)	7·22	330·0 (+65·0%)	+4·7 (+1·4%)
LP50%:	100 ml	−100·0 (−50·0%)	5·88	205·8 (+105·8%)	−119·4 (−36·7%)
Alb20%:	100 ml	0·0 (0%)	20·3	465·4 (+365·4%)	−291·2 (−62·6%)
Alb4%:	100 ml		4·12	174·2 (+74·2%)	
HES:	300 ml	‐	‐	‐	‐

^a^ Potential water recruitment: absolute product volume (ml) + (WBC_A_ (ml/g) x Albumin (g)).

^b^ ∆Potential volume load: PVL difference between conventional and volume‐reduced products.

^c^ FFP volume is 300 ml; however, equivolumetric doses were used to more accurately calculate PVL.

^d^ Calculated using maximum albumin range for RBCs (5·0 g/l)

### Effect of storage lesion on transfusion product oncotic pressure

During storage of cellular blood products (i.e. all red cell and platelet products), there was an expected change in metabolites, including a decrease in glucose and pH and an increase in lactate and haemolytic parameters (the latter only in RBC products). Albumin remains constant and only minimal increases in total protein are seen (Table 3). The difference in COP between fresh and maximally stored cellular blood products was negligible with a maximum increase of only 1·1 mm Hg (Fig. 2b).

**Table 3:** Storage of conventional and volume‐reduced products

Characteristics	RBC		VR‐RBC		PLT		VR‐PLT	
	Day 1 (*n* = 3)	Day 35 (*n* = 3)	Fresh (*n* = 3)	6 h (*n* = 3)	Day 1 (*n* = 3)	Day 7 (*n* = 3)	Fresh (*n* = 3)	6 h (*n* = 3)
COP (mm Hg):	1·86 (1·7–2·0)	2·55 (2·3–3·0)	1·45 (1·3–1·6)	1·47 (1·4–1·6)	7·53 (7·0–8·3)	9·2 (8·4–11·1)	7·82 (7·00–8·3)	7·71 (7·2–8·3)
Albumin (g/l):	<5·0	<5·0	<5·0	<5·0	13·2 (12·8–13·4)	13·4 (12·9–13·6)	12·9 (12·8–13·0)	13·2 (13·1–13·3)
Total protein (g/l):	3·18 (2·6–4·4)	3·83 (2·6–5·3)	3·37 (2·4–5·0)	2·61 (1·7–3·5)	23·33 (23·0–24·0)	24·00 (24·0–24·0)	22·33 (22·0–23·0)	23·33 (23·0–24·0)
Osmolality (mOsmol/l):	333·0 (332–335)	338·3** (337–340)	327·7 (326–330)	326·7 (324–328)	283·3 (252–300)	295·3 (279–306)	304·3 (303–306)	320** (318–321)
Cell count (cells/l•10^12^)	6·07 (5·5–6·4)	6·12* (5·5–6·5)	8·49 (8·2–8·7)	8·35* (8·2–8·6)	1·07 (0·97–1·2)	0·97* (0·86–1·14)	12·0 (10·6–13·4)	11·8** (10·3–13·2)
pH:	6·83 (6·78–6·89)	6·40** (6·39–6·42)	6·85 (6·82–6·89)	6·83 (6·81–6·87)	7·02 (7·01–7·04)	6·94 (6·89–6·99)	6·71 (6·68–6·73)	<6·0
Glucose (mmol/l):	33·3 (32·5–35·0)	18·6*** (17·5–20·2)	20·0 (19·5–20·6)	19·6*** (19·1–20·2)	8·7 (8·5–9·1)	3·1** (2·5–4·1)	10·4 (10·1–10·9)	2·3** (1·9–2·7)
Lactate (mmol/l):	4·96 (4·2–6·4)	26·1** (24·9–28·0)	4·7 (4·0–5·1)	5·4** (4·7–5·9)	3·4 (3·3–3·4)	12·8** (11·9–13·4)	5·6 (5·3–5·8)	23·3** (21·0–25·0)
fHb (mmol/l):	225 (201–269)	368 (288–414)	n·m·	n·m·	‐	‐	‐	‐
Haemolysis (%):	0·12 (0·11–0·15)	0·20* (0·16–0·22)	n·m·	n·m·	‐	‐	‐	‐

Data are displayed as mean (range).

Difference in biochemical properties at maximum storage duration vs. fresh product tested using paired‐sample Student's *t*‐test.

COP, colloid osmotic pressure; fHb, cell‐free haemoglobin; n.m., not measured; PLT, platelet concentrate; RBC, red blood cell unit; VR‐RBC, volume‐reduced red blood cell unit; VR‐PLT, volume‐reduced platelet concentrate.

* *P* < 0·05.

** *P* < 0·01.

*** *P* < 0·001.

### Correlation of measured COP with indirect measurements

**Fig. 3:**
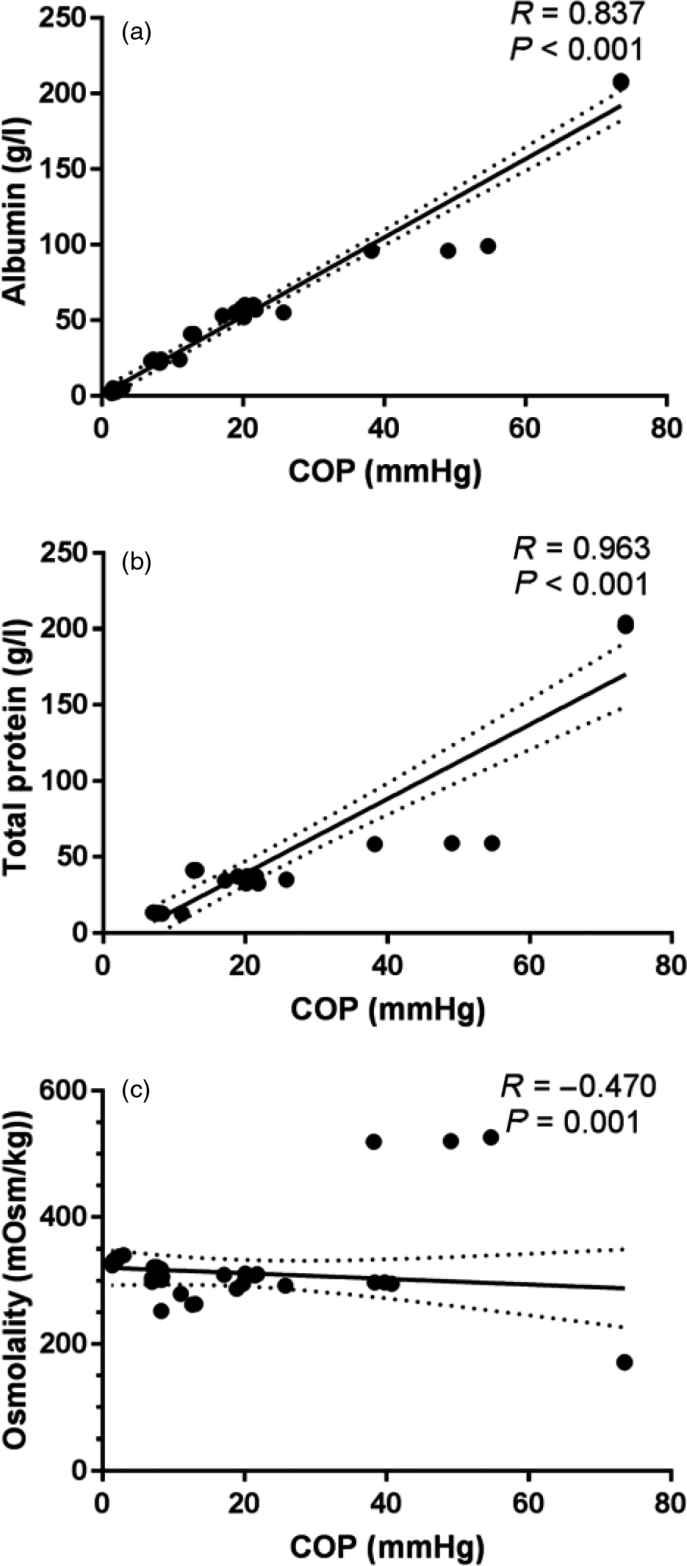
Correlation of indirect determinants and COP in blood products. Indirect determinants of COP are plotted against measured COP. Individual data points for fresh products with linear regression line and 95% confidence interval for slope are presented. Panels (a and b): Spearman's rank correlation tests show strong correlations between albumin, as well as total protein, and COP. Panel (c): Osmolality shows a negative correlation with COP which is still significant.

The primary determinants of COP include albumin, total protein (a combination of albumin, globulins and a small fraction of other proteins) and osmolality. There were strong correlations between COP and albumin (*R* = 0·83, *P* < 0·001) as well as total protein (*R* = 0·963, *P* < 0·001) (Fig. 3a‐b). Albumin was undetectably low using clinical measuring methods (<4 g/l) in RBC and VR‐RBCs and could therefore not be assessed. Product osmolality showed a moderate correlation to COP, though contrary to expectations the relation was inverse (Fig. 3c). Recurrent outliers were Alb20% and LP50%, the former relatively hypoosmolar compared with its COP and the latter hyperosmolar compared with its COP.

### Correlation of calculated with measured COP

**Fig. 4:**
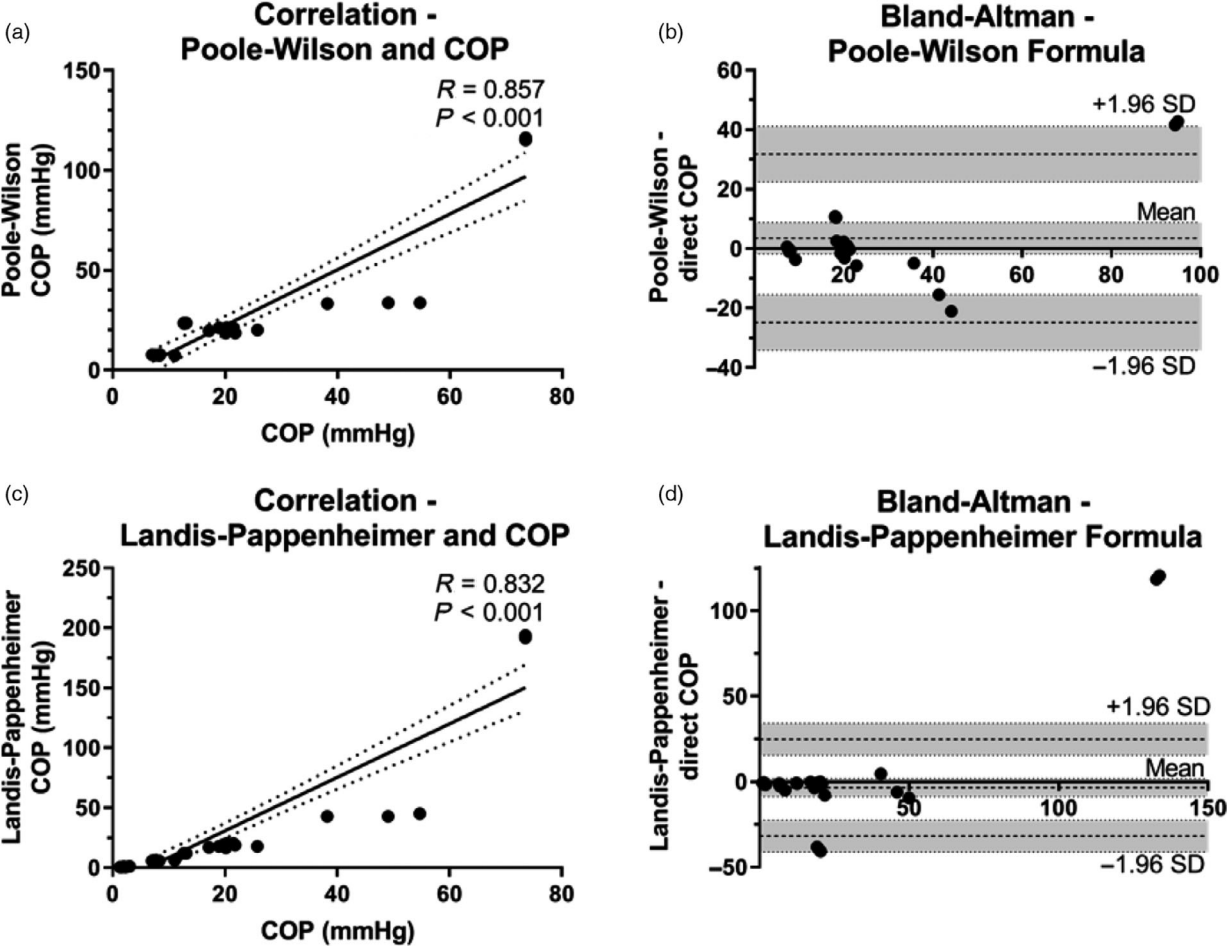
Correlation of calculated and directly measured COP. Calculated COP using two different formulas is plotted against directly measured COP. Individual data points for fresh products with linear regression line and 95% confidence interval for slope are presented. Panels (a and b): There was strong correlation between the Poole–Wilson (PW) formula and COP. Bland–Altman plotting shows good agreement with only Alb20% as outlier just outside the 95% confidence interval. Panels (c and d): There is a strong correlation between Landis–Pappenheimer calculated and directly measured COP. The agreement plot shows Alb20% far outside the outer confidence limit.

Two formulae were used to calculate COP: (1) Poole–Wilson formula and (2) Landis–Pappenheimer formula. Strong correlations were seen between calculated COP and directly measured COP (Fig. 4). Bland–Altman plots show that using both formulas that all values fall within the 95% agreement limits, only Alb20% is poorly calculable using these equations.

## Discussion

Colloid osmotic pressure is one of the principals of fluid homeostasis in the body [35], and COP of blood products could contribute to the development of TACO. This study investigated the COP of conventional and newer volume‐reduced products as well as the influence of storage lesion. The major findings of this study are as follows: (1) there is a wide variation in COP between transfusion products, with conventional RBCs, PLTs and FFP being hypooncotic; (2) volume reducing RBCs and PLTs does not increase COP, however partially reconstituted lyophilized plasma has a greatly increased COP; and (3) storage lesion does not result in relevant increases in COP.

### Blood products have low COP

We hypothesized COP could be a determining factor in TACO; however, conventional blood products are all hypooncotic relative to plasma COP. These results suggest that developing TACO after RBC or PLT transfusion is not driven by recruitment of volume through hyperoncotic transfusion products. A very low COP in RBCs was seen before by Hiipala *et al.* [36] The removal of plasma and addition of storage medium likely decrease COP for RBCs and PLTs, even though some storage solution including SAGM contain mannitol which is also osmotically active. Alternative mechanisms for TACO after RBC transfusion include NO scavenging due to cell‐free haemoglobin [37, 38]. Two recent studies of large cohorts show an increased pulmonary artery pressure (PAP) after transfusion of a single unit of RBCs in a critically ill population as well as patients after cardiac surgery [39, 40]. Increased PAP can indicate increased pulmonary capillary pressures, the first step in developing hydrostatic pulmonary oedema, that is TACO. Alternative mechanisms behind TACO are beyond the scope of this study but include in the case of RBC‐induced TACO: endothelial and/or glycocalyx damage due to inflammatory mediators, [41] increased blood viscosity [42] or plasma soluble mediators (though more likely in platelet and plasma transfusion compared with RBCs) [43].

The COP per product type matches the incidence pattern seen in TACO, the incidence being highest after FFP transfusion, followed by RBCs and PLTs [12, 13, 44]. However, transfusion products being hypooncotic make it unlikely that large volumes of extravascular fluid are recruited. Another explanation for FFP as highest risk product could be indication bias. FFP was long used for reversal of vitamin K‐antagonists in the absence of bleeding; however, the newest American Society of Hematology guidelines (2018) advise prothrombin complex concentrate over FFP [45]. Previously normovolemic, non‐bleeding patients would receive significant volumes of FFP (12–15 ml/kg body weight) for this indication, compared with a single‐unit transfusion policy of RBCs or PLTs transfused in the non‐emergent, non‐bleeding setting. A prospective study showed a dose‐dependent increase in TACO with the number of units FFP transfused [46]. Whether the COP and FFP association has a cause–effect relationship or whether confounders such as patient factors are involved require further research.

### Total volume load reduced by concentrated products

Volume reduction in RBCs and PLTs does not increase COP, while absolute volumes decrease (Table 3). To our knowledge, this study is the first to investigate the PVL of blood products, calculated for each of the products investigated using the WBC_Alb_ (Table 2). The ∆PVL of VR‐RBCs and VR‐PLTs and LP50% compared with conventional products is, respectively, −16·7%, −93·4% and −199·4%. Based on the decreased transfused volume as well as potential recruited volume, VR‐RBCs and VR‐PLTs will likely prevent TACO. Even in LP50% which has a very high COP, the negative ∆PVL compared with conventional FFP suggests it decreases volume load and possibly prevents TACO.

Transfusion of FFP, Alb4% and LP100% with near‐physiological COP levels is not likely to result in clinically significant volume recruitment. Therefore, TACO is unlikely an effect of massive volume recruitment. Protein, however, is retained intravascularly for a prolonged period – the effects of this are beyond the scope of this in vitro study and could still contribute to TACO.

### Effect of storage lesion on COP

Storage lesion did not significantly increase COP (Table 3). While metabolic products such as glucose significantly decrease, lactate levels increase and cell lysis occurs as reflected in cell counts and haemolysis, there is no significant increase in COP or total protein. While this study adhered to Dutch product storage guidelines of 35 days, due to only minor changes observed during storage we expect these factors to still not being clinically relevant if products are stored up to 42 days or even 49 days as is customary in some other countries.

Correlations between indirect determinants (i.e. albumin, total protein and osmolality) and COP were analysed, as well as Bland–Altman plots used to compare calculated and directly measured COP (Figs. 3and4). Even though strong correlations were found between indirect determinants and COP, the practical applicability is limited. The non‐linear relation found between COP and total protein between Alb4% vs. Alb20% and similarly LP50% vs. LP100% is likely explained by the Gibbs‐Donnan effect [47, 48]. Negatively charged proteins unable to cross the semi‐permeable barrier of the colloid osmometer result in an electric gradient and secondary osmosis effect. While measured as an increase in net‐osmotic force, this falsely elevates COP. The effect appears primarily relevant for products containing supraphysiologic protein concentrations. While a rough estimate of COP can be made based on surrogate measures, direct measurement is inexpensive and remains the gold standard.

This study has a number of limitations, first of all this is a small study with three units per product sampled which does not show the true spread of biochemical product characteristics. A large inter‐unit variation in COP cannot be ruled out. Additionally, blood product preparation differs between countries, that is the volume of residual plasma per product as well as the storage solutions added, both influencing COP. Furthermore, using a calculated PVL has a number of limitations. Albumin was used as determinant for water recruitment as COP is a vector and cannot be used to calculate volume changes, nor is there any literature describing total protein concentration on water binding capacity. Therefore, PVL by HES was not calculable and the PVL might be underestimated in the case of LP50% and Alb20% as COP increased non‐linear to albumin load. Finally, the recruited volume is dependent on the oncotic pressure of the patient. In hyperoncotic patients, for example in the context of dehydration, low COP products will result in relative dilution of plasma and less volume will be recruited. This is in sharp contrast to patient liver failure who is hypooncotic and plasma protein load relatively increases. Nevertheless, PVL is based on maximum volume recruitment independent on patient context and thereby could be useful as an ex vivo measure to compare a product effect on circulating volume.

To accurately evaluate the effect of COP on volume recruitment, clinical studies are required. Baseline plasma COP, post‐transfusion COP and that of the infusion product should be measured. As it is unknown exactly how long these products remain intravascularly, it is crucial to link these factors to circulating volume after, respectively, crystalloid and colloid infusion, and of course transfusion.

## Conclusion

Transfusion‐induced colloid osmotic fluid recruitment from the third space based on biochemical factors is unlikely as most products transfused have sub‐physiological COP levels. While additional protein infusion contributes to increased total intravascular protein levels, the calculated PVL is not greatly increased. Storage lesion does not significantly increase COP, and we calculated that volume‐reduced RBCs and PLTs products are likely to reduce intravascular transfusion volume, while this remains unsure in partially reconstituted lyophilized plasma.

## Sources of funding

The blood products used in this study were provided by Sanquin, the Dutch blood bank, as well as the German Red Cross Blood Service West, Hagen, Germany.

## Conflicts of interest

The authors declare no conflict of interests.

## Supporting information


**Table S1**. Transfusion products and measurements.
