# Supplementary material for: Colloid osmotic pressure of contemporary and novel transfusion products
Source: Vox Sang. 2020 May 6;115(8):664–75. doi: 10.1111/vox.12932 (PMC7754447; doi:10.1111/vox.12932)
Supplement: Supplementary file 1 — Table S1. Transfusion products and measurements. [file VOX-115-664-s001.docx]

**Supplementary Table 1. – Transfusion products and measurements**

|  | **Products:** | **Storage Duration:** | **Measurements:** | |
| --- | --- | --- | --- | --- |
| **RED BLOOD CELLS** | Red blood cells (RBC) | 1 day | Cellular^‡^:  Oncometry:  Blood gas:  Osmolality: | CBC, 2,3-DPG, ATP  COP, glucose, albumin, total protein  Na^+^, K^+^, pH, lactate  Freezing-point method |
|  |  | 35 days |  |  |
|  | Volume-reduced red blood cells (VR-RBC) | Fresh |  |  |
|  |  | 6 hours |  |  |
| **PLATE-LETS** | Platelet concentrate (PLT) | 1 day | Cellular^‡^:  Oncometry:  Blood gas:  Osmolality: | Platelet count, swirl sign  COP, glucose, albumin, total protein  Na^+^, K^+^, pH, lactate  Freezing-point method |
|  |  | 7 days |  |  |
|  | Volume-reduced platelet concentrates (VR-PLT) | 1 hour |  |  |
|  |  | 6 hours |  |  |
| **PLASMA** | Fresh frozen plasma (FFP) | N.A. | Oncometry:  Blood gas:  Osmolality: | COP, glucose, albumin, total protein  Na^+^, K^+^, pH, lactate  Freezing-point method |
|  | Lyophilized plasma (LP100%) | N.A. |  |  |
|  | Lyophilized plasma (LP50%) | N.A. |  |  |
| **COLLOID** | Albumin 40 g/L (Alb4%) | N.A. | Oncometry:  Blood gas:  Osmolality: | COP, glucose, albumin, total protein  Na^+^, K^+^, pH, lactate  Freezing-point |
|  | Albumin 200 g/L (Alb20%) | N.A. |  |  |
|  | Hydroxyethyl Starch 130/0.4 (HES)* | N.A. |  |  |

**: Tetraspan 6%®; CBC:* Complete blood count; *2,3-DPG:* 2,3-diphosphoglycerate; *ATP:* adenine triphosphate; *COP: Colloid osmotic pressure; N.A.: Not applicable*
